# A novel targeted NGS panel identifies numerous homologous recombination deficiency (HRD)-associated gene mutations in addition to known *BRCA* mutations

**DOI:** 10.1186/s13000-023-01431-8

**Published:** 2024-01-06

**Authors:** Anne Vogel, Anna Haupts, Michael Kloth, Wilfried Roth, Nils Hartmann

**Affiliations:** Institute of Pathology, University Medical Center Mainz, Langenbeckstraße 1, Mainz, 55131 Germany

## Abstract

Deleterious mutations in the *BRCA1* and *BRCA2* genes have significant therapeutic relevance in clinical settings regarding personalized therapy approaches. *BRCA1* and *BRCA2* play a pivotal role in homologous recombination (HR) and thus are sensitive for PARP inhibitors (PARPi). Beyond the narrow scope of evaluating only the *BRCA* mutation status, PARPi can be beneficial for HR deficient (HRD) patients, who harbor mutations in other HR-associated genes. In the present retrospective study, a novel targeted HRD gene panel was validated and implemented for use with FFPE tissue. Samples of patients with ovarian, breast, pancreatic and prostate cancer were included. Variants were robustly detected with various DNA input amounts and the use of test samples showed complete concordance between previously known mutations and HRD panel results. From all the 90 samples included in this cohort, *TP53* was the most frequently altered gene (73%). Deleterious *BRCA1/2* mutations were found in 20 (22%) of all samples. New pathogenic or likely pathogenic mutations in additional HR-associated genes were identified in 22 (24%) patients. Taken together, the present study proves the feasibility of a new HRD gene panel with reliable panel performance and offers the possibility to easily screen for resistance mutations acquired over treatment time.

Mutations in HR-associated genes, besides *BRCA1/2*, might represent promising potential targets for synthetic lethality approaches. Thus, a substantial number of patients may benefit from expanding the scope of therapeutic agents like PARPi.

**Supplementary Information:**

The online version contains supplementary material available at 10.1186/s13000-023-01431-8.

## Background

Over the past years, the clinical and therapeutic significance of evaluating the mutational status of genes accounting for homologous recombination deficiency (HRD) has increased. Various studies demonstrated the prevalence of HRD in multiple tumor types like ovarian, breast, pancreatic and prostate cancer [1–3]. Those findings suggest an increasing number of patients who could benefit from specific therapies of HR deficient solid tumors.

Homologous recombination (HR) is a cellular mechanism used to restore DNA following double strand breaks (DSB), which considers the original sequence through using a homologous sister chromatid as template. Several genes like *ATM*, *NBN*, *RAD50*, *PALB2* but especially *BRCA1* and *BRCA2* are involved in HR and represent targets for therapy. Defects and disrupted function in recombination genes result in the accumulation of mutations and in the necessity to rely on non-homologous end joining (NHEJ) for DSB repair. Repairing damaged DNA by NHEJ leads to fatal errors and subsequently to cell death [4–6].

Tumors deficient in HR are sensitive to platinum-based chemotherapy and inhibitors of Poly (ADP-ribose) polymerase (PARP). *PARP1* regulates the restoration of DNA by Base Excision Repair (BER). Normal cells are able to use both reparation mechanisms. Thus, due to dysfunctional proteins in those pathways, HR deficient tumor cells treated with PARP inhibitors (PARPi) subsequently accumulate lethal DNA defects referred to as synthetic lethality [7]. The cell is eventually no longer able to tolerate the damage, which results in cell death [8, 9].

In 2014, the U.S. Food and Drug Administration (FDA) approved Lynparza (olaparib) as the first PARP inhibitor in advanced ovarian cancer for maintenance therapy with germline mutations in *BRCA1* and *BRCA2* genes, which function as predictive biomarkers. In the following years, the FDA and the European Medicines Agency (EMA) approved additional PARPi naming rucaparib, niraparib, and talazoparib for treatment in several cancer types [8].

In following studies demonstrating the proficient effect of PARP inhibition in breast, prostate and pancreatic cancers, *PARP1* emerged as a new target for platinum-sensitive tumors, thereby helping personalized therapy and HRD testing in routine diagnostics to receive increased relevance and importance. In addition to defects in *BRCA1/2*, also loss-of-function mutations in *ATM*, *RAD50*, *PALB2* or other crucial HR genes equally may contribute to HR deficiency [4, 10, 11].

Mutations in *BRCA1/2* genes (BRCAm) were most frequently identified in ovarian (OvCa, 15.2%) followed by prostate (PC, 10.7%), breast (BC, 8.8%) and pancreatic cancer (PaC, 5.2%) [12]. But the HRD phenotype can also manifest independent of BRCAm in those cancer types with mutations in other HR associated genes that occur with a prevalence of 9.2% for OvCa [13], 11% for BC [14], 6% for PaC [15] and 16% for PC [16]. The consequences of HRD can be assessed at the DNA level by the identification of genomic scars resulting in a genomic instability score or HRD score.

Due to the FDAs original approval of olaparib based on the *BRCA1/2* status in ovarian cancer, current standard testing does not always include other HR genes, despite the fact that the FDAs approval for olaparib as treatment for castration-resistant PC was based on the findings of the PROfound trail which also included *ATM* as a predictive biomarker [17]. This demonstrates that further HR associated genes might be able to act as predictive biomarkers and therefore, more patients could benefit from therapy with PARPi or platinum-based chemotherapy.

The purpose of this retrospective study was to identify ovarian, breast, prostate and pancreatic cancer patients who may have additional mutations and were previously only tested for *BRCA1* and *BRCA2* but may benefit from PARPi. In total, 42 h-associated genes were included in the evaluation by means of a novel targeted next-generation sequencing (NGS) panel. Formalin-fixed, paraffin-embedded (FFPE) samples of 90 patients, which were previously screened for *BRCA1/2* mutations, were analyzed. Furthermore, including the analysis of additional genes in routine diagnostic testing for HRD may generate new insights into PAPRi resistance mechanisms and represents the basis of personalized oncology approaches.

## Materials and methods

### Patient selection

For validation, 24 formalin-fixed, paraffin-embedded (FFPE) samples with known mutation status of *BRCA1/2* or *ATM*, consisting of 11 ovarian, three breast cancer samples and ten colorectal liver metastases (CRLM) previously evaluated by whole exome sequencing [18] were used as a training cohort. In addition to those samples, a commercially available *BRCA* Somatic Multiplex FFPE standard (HD810, Horizon Discovery LTD., Group, Cambridge, UK) with 16 mutations in *BRCA1*, *BRCA2*, *BRIP1*, *BARD1* and *NBN* was included.

For the subsequent retrospective analysis of HRD prevalence, samples, which were tested for *BRCA1/2* mutations earlier were examined. Of those, 90 fit the inclusion criteria defined as follows: tumor cell count of at least 30%, sufficient DNA concentration and volume for an input of 100 ng that would allow for library preparation. The amount of tumor cells was quantitatively assessed by means of hematoxylin and eosin staining. Imitating the circumstances of a routine diagnostic setting, primary tumors and metastases of ovarian (n = 63), breast (n = 19), pancreatic (n = 9) and prostate (n = 5) cancer were included regardless of the histological subtype of the tumor. The local ethics committee (Ethics Committee of the Rhineland-Palatinate State Medical Association, Mainz, Germany) approved the study design and all samples were handled in compliance with the standards proposed by the Declaration of Helsinki.

### DNA extraction and quantification

Tumor-specific genomic DNA extraction from unstained tissue slides was performed using Maxwell RSC FFPE Plus DNA Kit (Promega, Madison, WI, USA) according to the manufacturer’s technical manual, with proteinase K digestion overnight. Quantification of DNA samples was conducted with Qubit DNA HS assay Kit (Thermo Fisher Scienfific, Waltham, MA, USA).

### Targeted next-generation sequencing

The QIAseq targeted DNA workflow (Qiagen, Hilden, Germany) was used to generate NGS libraries. The novel QIAseq panel includes 42 genes that are involved in DNA damage repair (*ARID1A*, *ATM*, *ATR*, *ATRX*, *BAP1*, *BARD1*, *BLM*, *BRCA1*, *BRCA2*, *BRIP1*, *CDK12*, *CHEK1*, *CHEK2*, *ERCC3*, *FAM175A*, *FANCA*, *FANCC*, *FANCD2*, *FANCE*, *FANCF*, *FANCG*, *FANCI*, *FANCL*, *HDAC2*, *MLH3*, *MRE11A*, *NBN*, *PALB2*, *PPP2R2A*, *RAD50*, *RAD51*, *RAD51B*, *RAD51C*, *RAD51D*, *RAD52*, *RAD54L*, *RNASEH2A*, *RNASEH2B*, *RNASEH2C*, *TP53*, *WRN*, *XRCC2*). The panel contains 799 amplicons with 1893 primer pairs and covers the complete coding sequence of all 42 genes and flanking intronic sequences. Typically, 100 ng genomic DNA was used as input and library preparation was performed according to the QIAseq Targeted DNA Panel Handbook. Briefly, in a multi-enzyme reaction, genomic DNA samples were fragmented, end repaired and A-tailed. Following sample indexing with unique molecular index (UMI), the targets were enriched by single primer extension. The final library was amplified by a universal primer.

For sequencing, the libraries were pooled equivalently, denatured and 8 million reads were assessed to one sample. The final loading concentration varied from 1.1 pM to 1.3 pM. Paired-end libraries were sequenced on an Illumiina NextSeq 500 with v2.5 reagent kit chemistry (Illumina, San Diego, Ca, USA) using 2 × 150 bp output with usage of a custom sequencing primer for read 1 (QIAseq A Read1 Primer I).

### Data analysis and variant calling

Generated bcl files were converted to FASTQ format and imported into the CLC Genomics Workbench version 12.0 (Qiagen Bioinformatics). This software was used to analyze and annotate sequenced samples on the basis of the human reference genome GRCh37 (hg19). Variant annotation was based on RefSeq accession numbers. The detection limit of variants was set to 10% allele frequency. Polymorphisms were excluded from further analysis by filtering against dbSNP. Furthermore, intron variants with potential splice effect were only taken into account if they were located one or two base pairs outside the exon. To ensure sufficient quality and sensitivity of the sequenced samples, only targets with a minimum coverage above 100 reads were considered. Targets with a read depth below 100x were excluded from the analysis.

### Data interpretation

The interpretation and classification of the detected variants was based on the guidelines of the American College of Medical Genetics and Genomics (ACMG) [19]. Pathogenicity was assessed by the five-tier system of classification: [1] pathogenic, [2] likely pathogenic, [3] uncertain significance, [4] likely benign, or [5] benign. Detected variants, which lead to a premature stop codon, a frameshift in the open reading frame or disruption of the splice site, were classified as likely pathogenic and therefore included in the tier 2 category. Remaining variants of uncertain significance (VUS) with no entry in the common databases were submitted to the prediction tools SIFT [20] and CADD [21] for functional analysis. Based on the predicted result, VUS were processed as likely pathogenic or likely benign. All Variants in tier 4 or 5 were dismissed and excluded from further analysis.

## Results

### Validation and implementation of a custom next generation sequencing panel for selected HRD associated genes

All 42 genes included in this panel play a pivotal role in HR or stand in close functional relation to the DNA repair pathway [22, 23]. Genes included in this panel were chosen based on publications listed in Supplementary Table S1.

To implement and assess the sequencing output of the new HRD panel, previously analyzed samples and a *BRCA* Somatic Multiplex FFPE standard were used. Only tumour samples with > 30% tumor cellularity were included and only mutations with > 10% frequency were considered. Large-scale deletions of several hundred or thousand base pairs cannot be identified with the current NGS panel. After initial sequencing, it became apparent that the coverage in specific regions was insufficient throughout all samples. In order to allow for appropriate evaluation of the variance with high predictive confidence, a cut-off coverage of at least 100 reads per target region was applied for the HRD panel. Therefore, a booster panel (containing 34 primers) was designed, which improved the coverage in those specific areas and increased the number of included primers from originally 1859 to 1893 (Supplementary Fig. S1).

The optimal DNA input amount was determined with a *BRCA* Somatic Multiplex FFPE standard by using decreasing amounts of gDNA from 100 ng to 5 ng as input for library preparation. Here, the detection limit was reached at 15 ng DNA input as one variant was undetectable (Supplementary Fig. S2). Thus, 100 ng of DNA was used as input for HRD library preparation in further analyses.

Variant detection performance was evaluated by 24 samples of the training cohort with known mutations in either *BRCA1/2* or other HR-associated genes. Comparative analyses of the previous results and the HRD panel outcome showed complete concordance regarding variant detection throughout all samples (Supplementary Fig. S3). Those corresponding findings concluded the validation and implementation process of the new panel.

### Effective mutation analysis with the new HRD panel

In this retrospective analysis, 98 patients were included who were previously analyzed for *BRCA1/2* status (Table 1). In total, eight samples had to be excluded after sequencing (dropout rate 8.2%) due to poor DNA quality and, consequently, insufficient sequencing performance and target sample coverage. Hence, the following molecular pathological analysis with the new HRD panel consisted of 90 samples including 14 from the 24 validation samples. The main and therefore most meaningful sample size was made up by ovarian carcinomas (n = 63).

**Table 1:** Patient cohort characteristics

	Prostate cancer (PC)	Pancreatic cancer (PaC)	Breast cancer (BC)	Ovarian cancer (OvCa)
**Mean age at sample collection in years (range)**	60 (41–72)	59 (33–74)	56 (34–75)	64 (27–84)
**Sex**				
**Male [n (%)]**	5 (100%)	5 (56%)	0 (0%)	0 (0%)
**Female [n (%)]**	0 (0%)	4 (44%)	13 (100%)	63 (100%)
**Mutation status**				
**BRCA1/2 mut**	0 (0%)	0 (0%)	4 (31%)	16 (25%)
**wild type**	5 (100%)	9 (100%)	9 (69%)	47 (76%)

**Fig. 1:**
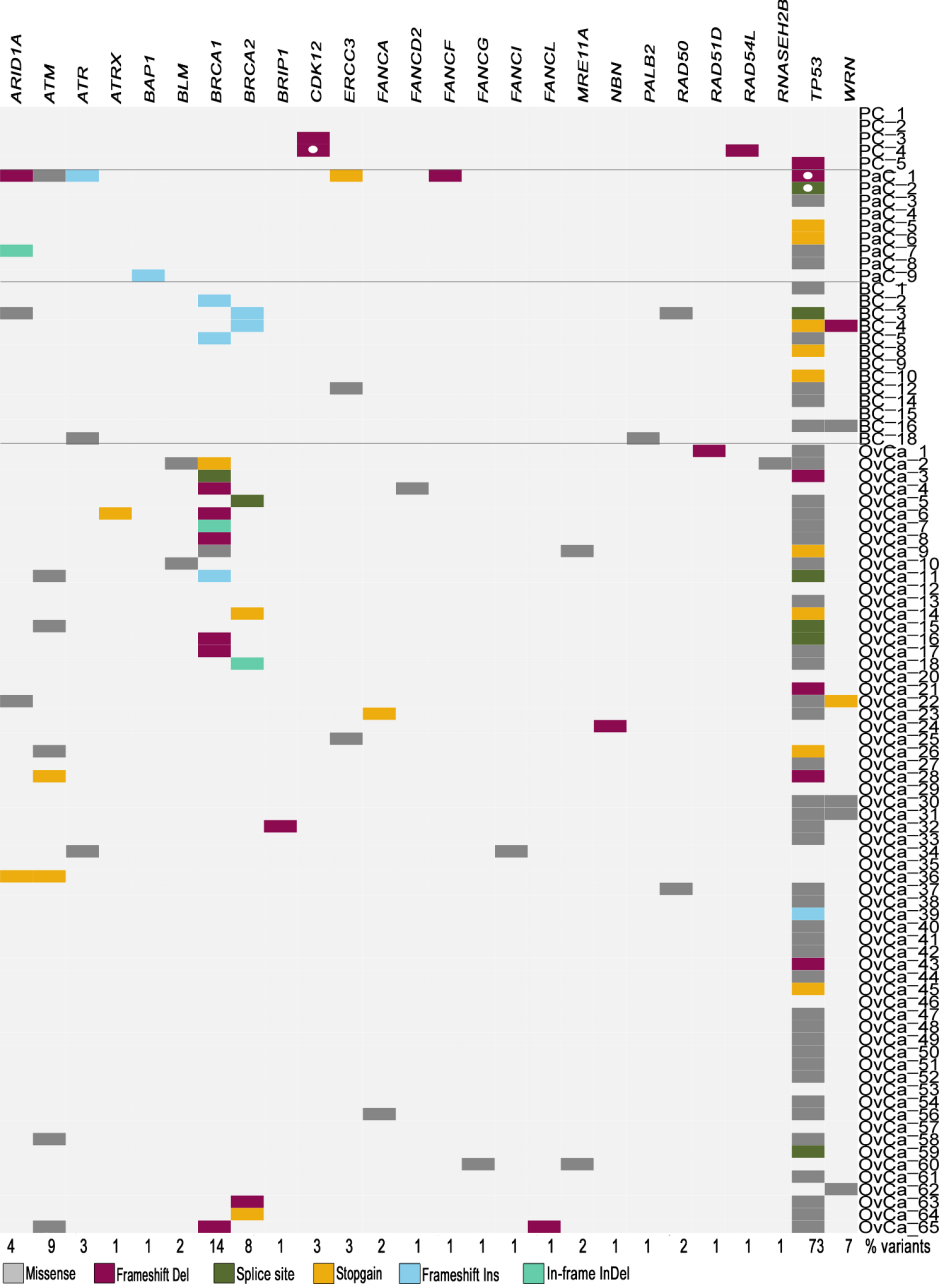
Heatmap of all variants found by means of the HRD panel. Investigated samples (n = 90) include prostate (n = 5, PC), pancreatic (n = 7, PaC), breast (n = 13, BC) and ovarian cancers (n = 63, OvCa) and are arranged according to their tumor type. All HRD genes with at least one variant are included and listed in the first row. Squares incorporating a white dot symbolize an additional variant in this gene. Below, the mutation rate in all samples per gene is shown in percent. Colored rectangles represent different variant classes: grey = missense variants, deep pink = deletions that lead to a frameshift in the open reading frame, olive = variants effecting the splice site, yellow = variants that lead to a premature stop codon, light blue = insertions that lead to a frameshift and aquamarine = deletions or insertions that do not lead to a frameshift. Only pathogenic, likely pathogenic and variants that were predicted likely pathogenic are presented. Likely benign or benign variants were excluded from further analysis

The heatmap (Fig. 1) only shows likely pathogenic or pathogenic alterations and their biological consequences. A detailed list of all identified mutations is provided in Supplementary Table S2. Mutations in the gene *TP53* were most abundant and accounted for 73% (n = 66) of modifications throughout all samples. Besides previously detected *BRCA1/2* variants (22%), *ATM* was the second most altered gene (9%). In *BRCA1/2* mutated samples, additional variants, were identified in eight cases (BC_3, BC_4, OvCa_2, OvCa_4, OvCa_6, OvCa_9, OvCa_11 and OvCa_65). In prostate cancer, deleterious variants were found in three out of five samples. Three samples of pancreatic cancer (n = 9) presented variants in genes other than *TP53*. In ovarian cancer deletions, which lead to a frameshift in the open reading frame, occurred in potentially targetable HR genes *RAD51D* (OvCa_1), *NBN* (OvCa_24), *BRIP1* (OvCa_32) and *FANCL* (OvCa_65), besides deletions in *BRCA1/2* and *TP53*.

**Fig. 2:**
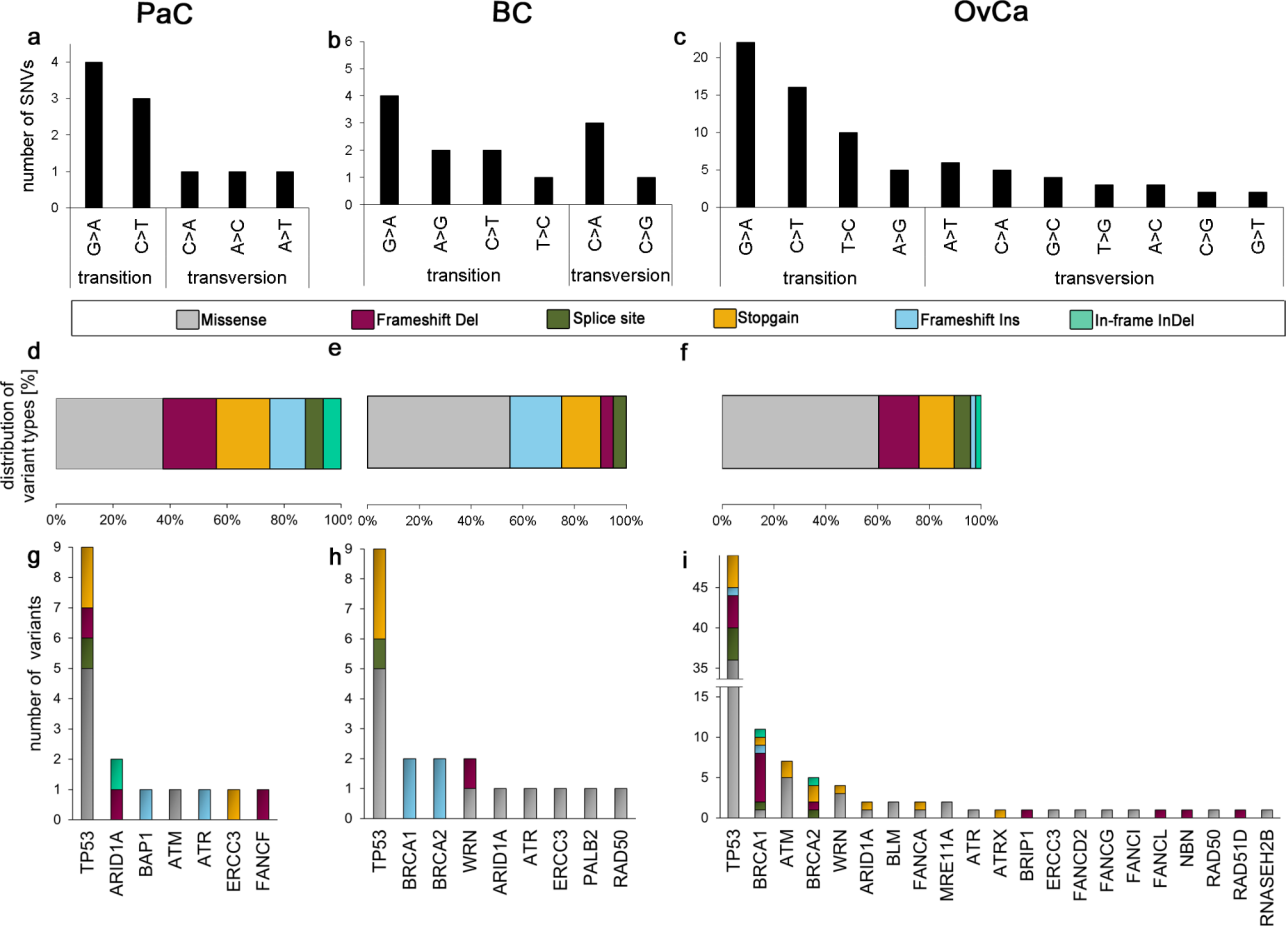
Summary of SNV class and variant distribution per tumor entity. The number of variant classes (a-c), variant distribution in percent (d-f) and the number of variants per gene (g-i) are shown for pancreatic (PaC: a, d, g (n = 7)), breast (BC: b (n = 10), e, h (n = 11)) and ovarian (OvCa: c (n = 53), f, I (n = 58)) cancer samples. Samples of prostate cancer are excluded from variant categorization due to the limited number of alterations found. Only pathogenic, likely pathogenic and variants that were predicted likely pathogenic are incorporated in this chart

Missense variants accounted for 54% of all alterations found in this study. Deletions, which lead to a frameshift in the open reading frame represented the second most abundant variants (17%); closely followed by stopgain variants (14%). The variant distribution was further investigated in Fig. 2 for pancreatic (a + b), breast (c + d) and ovarian (e + f) cancer samples. Most variants per tumor entity were detected in *TP53*. In ovarian cancer samples, 15% of all detected variants were deletions leading to a frameshift. Of all filtered SNVs, transitions were outweighed by transversions resulting in a ratio of transitions to transversions of 2.33 (pancreatic cancer), 2.25 (breast cancer) and 2.36 (ovarian cancer) with G > A being the most frequent base substitution in all entities.

All analyzed samples of this study are summarized in Table 2 and assigned to categories based on the detected variants. In addition to 20 (22.2%) patients with mutations in *BRCA1/2*, 22 (24.4%) of this cohort harbored mutations in additional HRD-associated genes, which were classified as pathogenic (tier 1) or likely pathogenic (tier 2) based on ACMG guidelines. Variants of unknown significance (VUS), which lead to a premature stop codon, a frameshift in the open reading frame, or disruption of the splice site, were classified as likely pathogenic and therefore included in the tier 2 category. The remaining VUS were evaluated by prediction tools and categorized accordingly. This subsequently lead to 5.6% of variants (5 patients), which were predicted as damaging, and 12.2% of variants (11 cases) with no significant effect on the protein function. Furthermore, 32 (35.6%) of patients have no indication of a HR dysfunction and present likely benign/benign variants and wild type sequences in the genes evaluated. Taken together, our data reveals that 22 (24.4%) of all 90 patients, who did not harbor a *BRCA1/2* mutation, exhibit deleterious variants in HRD-associated genes. Those findings highlight that a substantial amount of previously *BRCA1/2* WT patients were then identified as potential HRD-positive, which might be relevant for PARPi therapy. In total, 42/90 (48%) patients had tumors with mutation in at least one HR gene.

**Table 2:** Result summary of all investigated samples

	*BRCA1/2* variants	HRD-associated variants				*TP53* variants
	likely pathogenic/ pathogenic	likely pathogenic/ pathogenic	VUS (predicted damaging)	VUS (predicted tolerated)	likely benign/ benign + wild type	likely pathogenic/ pathogenic
**Ovarian Cancer (n = 63)**	16 (25.4%)	15 (23.8%)	4 (6.3%)	7 (11.1%)	21 (33.3%)	49 (77.8%)
**Breast Cancer (n = 13)**	4 (30.8%)	3 (23.1%)	0 (0%)	2 (15.4%)	4 (30.8%)	9 (69.2%)
**Pancreatic Cancer (n = 9)**	0 (0%)	2 (22.2%)	1 (11.1%)	1 (11.1%)	5 (55.6%)	7 (77.8%)
**Prostate Cancer (n = 5)**	0 (0%)	2 (40.0%)	0 (0%)	1 (20.0%)	2 (40.0%)	1 (20.0%)
**Total (n = 90)**	**20 (22.2%)**	**22 (24.4%)**	**5 (5.6%)**	**11 (12.2%)**	**32 (35.6%)**	**66 (73.3%)**

## Discussion

Over the last decade, it has become apparent that both, germline and somatic, deleterious mutations in *BRCA1/2* (BRCAm) have disruptive effects on DNA damage repair by HR mechanisms and might subsequently be susceptible to PARP inhibition (PARPi) [4, 22, 24]. Especially patients with germline or somatic variants in HR-associated genes with functional *BRCA1/2* represent a group of clinical significance, which is disregarded by the narrow scope of solely determining BRCAm status. Different approaches (mutational and methylation analysis, genomic scar identification, functional assays, and combined tests) of assessing the HRD status are currently used in clinical settings to in order broaden the patient population who might benefit from PARPi therapy [25]. ESMO recommendations for HRD testing in ovarian cancer describe good clinical validity for BRCAm analysis and genomic scar assays [25]. The Myriad myChoice test (Myriad Genetics, Salt Lake City, UT) is the only test approved by the FDA for therapy with PARPi (olaparib and niraparib) in ovarian cancer and calculates an HRD score to define genomic scares and genomic instability by loss of heterozygosity (LOH), telomeric allelic imbalance (TAI), and large-scale state transitions (LST) [26]. Another approach by Foundation Medicine is to determine HRD by using genome-wide LOH as a biomarker in association with *BRCA1/2* [12]. Based on mutational signatures (somatic substitution, insertion/deletion, and rearrangement patterns) as well as genomic scars, HRDetect is an additional assay to predict *BRCA1/2* deficiency and was tested successfully in independent cohorts of patients with BC, OvCa and PaC [27]. Apart from that, the detection of *RAD51* nuclear foci correlates with PARPi sensitivity and is also a feasible method to predict the response to PARPi beyond *BRCA1/2* pathogenic variants in BC. The lack of *RAD51* foci formation is associated with the sensitivity to PARPi and distinguishes between responsive and resistant tumors with an improved predictive power compared to the HRD score [28, 29]. Standardization of HRD evaluation could be useful to ensure comparability as currently no gold standard for HRD assessment has been specified and approaches used in clinical practice are heterogeneous.

This study evaluated HRD status by targeted NGS for 42 h-associated genes. However, this technique does not allow for assessment of LOH, TAI and LST or the distinguishing between somatic and germline variants. There is an ongoing discussion about the contribution of mutations in non-BRCA HR-associated genes to the HRD status determined by genomic scars. Recent studies found no, little or good correlation between HRD status determined by mutations in non-BRCA HR-associated genes and determined by genomic scars [30–32]. In future, it will be important to define those genes and specific mutations that positively contribute to the HRD status. Here, the new HRD gene panel was validated and implemented under routine conditions and a low dropout rate of 8.2% was observed. All previously reported variants were detected in the validation process by two independent methods in FFPE samples and reference material.This proves the usability and feasibility of the new HRD panel in practice.

Due to the fact that ovarian cancer was the first cancer to be approved for PARPi therapy by the FDA in 2014 [33], ovarian cancer (OvCa) contributed the largest sample size. In 2018, 2019, and 2020 olaparib was approved for breast cancer (BC) [34], pancreatic (PaC) [35] and prostate cancer (PC) [22], respectively. Now, a variety of PARPi are approved in OvCa (olaparib, rucaparib and niraparib), BC (olaparib and ralazoparib), PaC (olaparib) and PC (olaparib and rucaparib) [36]. Thus, the cohort in this study included breast, ovarian, pancreatic and prostate cancer. The majority of samples were ovarian cancers, because one inclusion criteriion was the known BRCAm status. Unexpectedly, this cohort exhibited a high prevalence of mutations in HR-associated genes (46.6%). Among these samples, the BRCAm rate was 22.2% (20 patients) and an additional 24.4% (22 patients) harbored mutations in other HR-associated genes. In OvCa, *BRCA1/2* mutation rate was reported to be around 13–20% [1, 13] but those numbers vary widely (8.1 − 47.7%) depending on the histological subtype and the method used for HR evaluation [37]. For BC, BRCAm rate was reported to be 7% and the accumulated HRD prevalence was 11% [14]. The proportion of BRCAm to HR mutated genes, besides *BRCA1/2*, for pancreatic and prostate cancer was 5–6% and 4–16%, respectively [15, 16]. According to a different study, mutations in *BRCA1/2* were most commonly detected in ovarian (15.2%), prostate, (10.7%) breast (8.8%) and pancreatic cancer (5.2%) [12]. Another Pan-cancer study described a combined HRD frequency of 85% for OvCa, BC, PC, PaC. The landscape of HRD was also affected by tumor type, status of metastasis and cancer stage [38]. In in the present study, the samples resemble a heterogeneous cohort (including primary tumors, metastases and pre-treated patients) which is common under routine circumstances. Taken this into consideration, the data collected in this study are consistent with the current literature. Whether all 22 (24.4%) patients, who harbor at least one mutation in HRD-associated genes besides *BRCA1/2*, are susceptible for PARP inhibition needs to be determined. Furthermore, several HR associated genes (*ATM*, *ATR*, *NBN*, *RAD51*, *RAD53*, *CHEK1*, *CHEK2*, *FANC* genes, CDK12, *FAM175A* and *BARD1)* have been identified to contribute to synthetic lethality in combination with *PARG or PARP* inhibitors in vitro [39–42]. Thus, genes that were frequently mutated in this cohort were evaluated in the following regarding their significance in HR and therapeutic relevance.

Throughout all 90 patients included in the present study, *TP53* was the most frequently altered gene and was mutated in 73% of tumors. The *TP53* gene is the most commonly mutated gene in several types of human cancer [43, 44] and is therefore of constant interest in research projects. Although, *TP53* might be a relevant predictor in treatment response to PARPi because mutations are able to interfere with *ATM* function [45] and functional *TP53* might be necessary for PARPi effectiveness [46], there is currently no approved targeted therapy for tumors harboring *TP53* mutations.

In the present study, two *CDK12* mutations and one deleterious variant in *RAD54L* were detected in patients with PC. Those genes are used as predictive biomarkers in the PROfound phase 3 study, which the FDAs approval for olaparib was based on, with PARPi in castration-resistant PC [11]. The PROfound study compared olaparib as monotherapy to physician’s choice (enzalutamide or abiraterone). Overall, 27.9% of patients were identified as HR deficient and significant beneficial effects of PARPi concerning progression free survival were reported. Whereby patients with mutations in *BRCA1/2* and *ATM* showed the best response [47]. Patients with variants in *CDK12* or *RAD54L* had a better calculated survival, suggesting a beneficial effect of PARPi [17]. Another study in primary PC used HRD score to define genomic instability and reported a significantly higher score in tumors with germline *BRCA2* mutations than in *ATM* and *CHEK2* altered cases [48]. However, the evidence of a beneficial effect of PARPi in patients with *CDK12* mutations is more ambiguous. A recent study investigated the responsiveness of patients with loss-of-function *CDK12* mutations regarding PARPi and *PD-1* inhibitor (programmed cell death receptor-1) and detected no beneficial effect with PARPi on PSA response or objective response, but patients might be sensitive to immunotherapy treatment with *PD-1* [49].

In the present cohort, patients with at least one mutation in *ARID1A*, *ATM* and *ATR* represent a substantial subgroup and account for 13.3% of all patients. The signaling of *ARID1A*, *ATM* and *ATR* interact and affect each other [50–53] and implicate that defects in those genes are targetable for a novel lethal synthetic approach in patient treatment [50, 51]. In many cancer types, mutations in *ARID1A* are the most common among genes associated with HR and were reported to account for 6.4% in OvCa, 3.7% in BC, 5.5% in PaC and 0% in PC of mutations found in a retrospective study of 21 cancer lineages [1]. In the present study, two mutations of *ARID1A* each were detected in PaC and OvCa. Several studies have shown that loss of *ARID1A* sensitized tumors to PARPi therapy. This effect was enhanced when tumors presented additional defects in *ATR* function [51] or were pre-treated with ionizing radiation [53]. The ongoing phase 2 study ATARI is investigating the impact of olaparib (PARPi) monotherapy and in combination with an *ATR* inhibitor drug (AZD6738) in gynecological cancers and compares tumors with and without *ARID1A* loss (NCT04065269). An additional study of AZD6738 investigates solid tumors (including pancreatic cancer) and determines response rates to therapy with *ATR* kinase inhibitor monotherapy and in combination with olaparib in an *ATM* loss and an *ARID1A* mutated subgroup (NCT03682289). Another *ATR* inhibitor (M4344) in combination with PARPi (niraparib) is investigated in PARPi resistant ovarian cancer in a clinical trial (NCT04149145). However, the outcomes of those studies are yet to be determined. In cells harboring *ATM* mutations, PARPi or *ATR* inhibitors enhanced the apoptosis rate of pancreatic cancer and subsequently increased synthetic lethality [54]. In this study, damaging *ATR* variants were found in pancreatic, breast and ovarian cancer in one patient each. Furthermore, pathogen variants in *ATM* were the third leading mutation after *TP53* and *BRCA1* and were detected in 9% of all patients. Contradictory to the reported frequencies of *ATM* mutations in other studies (OvCa = 1.5% [1, 13], BC = 4% [14], PaC = 5% [16] and PC = 4% [15]), the present one found that *ATM* variants were most common in OvCa (n = 7) and absent in PC and BC, which can be explained by the limited overall sample size. In *ATM* deficient cell lines, monotherapy of PARPi (olaparib) showed proficient results and lead to reduction of viability but the apoptosis rate was significantly increased in combination with *ATR* inhibitors [54–57].

Moreover, 14% of the patients included in this study harbored deleterious mutations in Fanconi anemia genes (*FANCA*, *FANCD2*, *FANCF*, *FANCG FANCI* and *FANCL*) and in *WRN*, which have been reported to interact with *BRCA1* [58–60]. Furthermore, substantial crosstalk between *WRN* and *PARP1* is described [61]. In patients with triple negative BC, a selection of Fanconi anemia genes, amongst other genes, are used as biomarkers in an ongoing phase 2 clinical trial with PARPi (Talazoparib) (NCT02401347). Fanconi anemia genes and also *WRN* are subject to another phase 2 trial in germline mutated metastatic BC, where the response of patients with mutations in those genes is compared to the response of patients with mutations in *BRCA1/2*, *PALB2* and *CHEK2* in regard to combination therapy of immunotherapeutic agent *PD-1* (HX008) and PARPi (niraparib) (NCT04508803). All genes included in this study were reported to impact HR function but the relevance of some genes has recently been challenged.

Contradictory to the theoretical assumption, about 40% of patients with *BRCA1/2* deficient tumors fail to respond do PARPi monotherapy [62, 63] or acquire resistance over the course of application time [64, 65] revealing that HRD status is a very dynamic phenotype. Mechanisms of PARPi resistance include reversion or secondary mutations which restore protein expression, enhanced stabilization and protection of the replication fork (upregulation of *PARG*) and inhibiting NHEJ factors (downregulation of the SHIELDIN complex, *53BP1* and *REV7*). Those mechanisms lead to restoration of HR and subsequently promoting PARPi resistance [66–70]. Secondary or reversion mutations restoring HR competence, and therefore PARPi resistance, are most commonly described in *BRCA1/2* but are also reported in *RAD51C* and *RAD51D* [70, 71]. Therefore especially for patients with PARPi resistance, combination therapy with agents inhibiting *ATM*, *ATR*, *PARG* or immune checkpoints offer a substantial benefit in treatment response to overcome resistance mechanisms and reverse sensitivity to PARPi [51, 55, 56, 72]. The new 42 HRD gene panel allows for screening a large number of HR associated genes for the detection of primary, reversion and secondary mutations in genes besides *BRCA1/2*. Those analyses allow for targeting acquired resistance mutations, guide therapy decisions and may explain reverse sensitivity to PARPi resistance mechanisms on a genetical level. By monitoring a broad number of genes involved in HR, these findings may be particularly relevant in generating new insights into PAPRi resistance mechanisms and may help to guide treatment decisions in order to adapt personalized therapy of patients.

In summary, the results of the present study show the feasibility of the new 42 HRD gene panel and reflect the prevalence of HR-associated genes in routine conditions. The NGS approach detected variants with complete concordance resulting in reliable panel performance. Characteristic HR phenotype was present in each cancer entity and was heterogeneous in that way. BRCAm was shown to account only for a subgroup of HRD tumors. Although *BRCA1/2* HR-phenotype claims to achieve most proficient effects with PARPi therapy, other genes manifest to be promising in some (pre-) clinical settings; especially in combination with other agents like *ATR* inhibitors or immunotherapy. However, the conclusive clinical relevance and impact in treatment strategy needs to be determined by further research. In particular, in tumors harboring mutations in uncommon HR-associated genes or VUS, the contribution to synthetic lethality is still uncertain. Lastly, our data highlight the importance of further evaluating HRD genes and addressing PARPi resistance mechanisms, beyond *BRCA1/2*, in clinical settings to expand the scope of therapeutic approaches with PARPi or combination therapies to refine treatment selection for optimal personalized therapy in cancer patients.

### Electronic supplementary material

Below is the link to the electronic supplementary material.

Supplementary Material 1

## Data Availability

The datasets generated during and/or analyzed during the current study are available from the corresponding author on reasonable request.

## Abbreviations

ACMG: American College of Medical Genetics and Genomics
BC: Breast cancer
BER: Base Excision Repair
BRCAm: BRCA mutations
DSB: Double strand breaks
EMA: European Medicines Agency
FDA: U.S. Food and Drug Administration
FFPE: Formalin-fixed paraffin-embedded
HR: Homologous recombination
HRD: Homologous recombination deficiency
LOH: Loss of heterozygosity
LST: Large-scale state transitions
NGS: Next-generation sequencing
NHEJ: Non-homologous end joining
OvCa: Ovarian cancer
PaC: Pancreatic cancer
PARP: Poly (ADP-ribose) polymerase
PARPi: PARP inhibitors
PC: Prostate cancer
TAI: Telomeric allelic imbalance
VUS: Variants of uncertain significance
WES: Whole exome sequencing
WGS: Whole genome sequencing
