# Supplementary material for: A novel targeted NGS panel identifies numerous homologous recombination deficiency (HRD)-associated gene mutations in addition to known BRCA mutations
Source: Diagn Pathol. 2024 Jan 6;19:9. doi: 10.1186/s13000-023-01431-8 (PMC10770950; doi:10.1186/s13000-023-01431-8)
Supplement: Supplementary file 1 — Supplementary Material 1 [file 13000_2023_1431_MOESM1_ESM.docx]

**A novel targeted NGS panel identifies numerous homologous recombination deficiency (HRD)-associated gene mutations in addition to known *BRCA* mutations**

Anne Vogel^1^, Anna Haupts^1^, Michael Kloth^1^, Wilfried Roth^1^, Nils Hartmann^1*^

^1^Institute of Pathology, University Medical Center Mainz, Langenbeckstraße 1, 55131 Mainz, Germany

**Supplementary Tab. S1**: Summary of all 42 genes included in the HRD panel and their contribution to DNA repair

| **Gene** | **Full Name** | **Contribution to DNA repair** |
| --- | --- | --- |
| *ARID1A* | AT-rich interactive domain 1A | interaction with *ATR*, recruited to DSBs (1) |
| *ATM* | ataxia telangiectasia mutated | recognition of DSBs, DNA resection on DSBs (2) |
| *ATR* | ataxia telangiectasia and Rad3-related | localization to sites of DNA damage (3) |
| *ATRX* | α-thalassaemia/mental retar- dation syndrome X-linked | chromatin remodeler, formation of sister chromatid exchanges (4) |
| *BAP1* | *BRCA1* associated Protein 1 | chromatin remodeling, controls the accumulation of other DNA-repair proteins (*BRCA1*, *RAD51*) (5) |
| *BARD1* | *BRCA1* associated RING domain protein 1 | interacts with *BRCA1*, DNA resection on DSBs(6) |
| *BLM* | Bloom syndrome protein | DNA resection on DSBs (7) |
| *BRCA1* | Breast cancer type 1 gene | interacts with *BARD1*, DNA resection on DSBs (2) |
| *BRCA2* | Breast cancer type 2 gene | localization of DSBs with *RAD51* and *PALB2* (2) |
| *BRIP1* | *BRCA1* interacting protein C-terminal helicase 1 | interaction with *BRCA1*, interstrand crosslink repair (8) |
| *CDK12* | Cyclin-dependent kinase 12 | regulation of expression of DNA damage response genes (9) |
| *CHEK1/2* | Serine/threonine-protein kinase Chk1/2 | transducer kinase of *ATM* (10)/ *ATR* (10) |
| *ERCC3* | General transcription and DNA repair factor IIH helicase subunit XPB | nucleotide excision repair (11) |
| *FAM175A* | *BRCA1*-A complex subunit Abraxas 1 | mediation of *BRCA1* recruitment (12) |
| *FANCA* | Fanconi anaemia complementation group A | interstrand crosslink repair, interaction with *BRCA1* (8,13) |
| *FANCD2* | Fanconi anaemia complementation group D2 | replication fork protection (8,13) |
| *FANCC/E/F/G/I/L* | Fanconi anaemia complementation group C/E/F/G/I/L | interaction with *FANCD2* (8,13) |
| *HDAC2* | Histone deacetylase 2 | interacts with *ATR*, chromatin remodeling (14) |
| *MLH3* | mutL homolog 3 | mismatch repair (15) |
| *MRE11A*, *NBN*,*RAD50* | meiotic recombination 11 homologue A, Nijmegen breakage syndrome 1, DNA repair protein *RAD51* homolog 1 | MRN complex detection and binding of broken DNA ends (2) |
| *PALB2* | partner and localizer of *BRCA2* | localization of DSB with *RAD51* and *BRCA2* (2) |
| *PPP2R2A* | Serine/threonine-protein  phosphatase 2 regulatory subunit B alpha | *ATM* phosphorylation (16) |
| *RAD51* | DNA repair protein *RAD51* homolog 1 | localization of DSBs with *BRCA2* and *PALB2*, invasion of DNA double helix (2) |
| *RAD51B/C/D*  *XRCC2* | DNA repair protein *RAD51* homolog 2/3/4, X-ray repair cross complementing 2 | forms BCDX2-Komplex, catalyze strand exchange(17) |
| *RAD52* | DNA repair protein *RAD52* homolog | interaction with *RAD51*, DNA resection on DSBs (18) |
| *RAD54L* | DNA repair and recombination protein *RAD54*-like | interaction with *RAD51*, strand invasion (18) |
| *RNASEH2A/B/C* | Ribonuclease H2 subunit A/B/C | removes incorrect ribonucleotides from DNA (19) |
| *TP53* | Tumor protein p53 | interaction with *RAD51*, strand invasion (18) |
| *WRN* | Werner syndrome gene | maintaining genomic stability, contributes to DNA repair, NHEJ repair pathway (20) |

1. Heeke AL, Pishvaian MJ, Lynce F, Xiu J, Brody JR, Chen W-J, et al. Prevalence of Homologous Recombination–Related Gene Mutations Across Multiple Cancer Types. JCO Precis Oncol. 2018;2(2):1–13.

2. Lord CJ, Ashworth A. BRCAness revisited. Nat Rev Cancer. 2016;16(2):110–20.

3. Maréchal A, Zou L. DNA damage sensing by the ATM and ATR kinases. Cold Spring Harb Perspect Biol. 2013;5(9):1–18.

4. Juhász S, Elbakry A, Mathes A, Löbrich M. ATRX Promotes DNA Repair Synthesis and Sister Chromatid Exchange during Homologous Recombination. Mol Cell. 2018;71(1):11-24.e7.

5. Yu H, Pak H, Hammond-Martel I, Ghram M, Rodrigue A, Daou S, et al. Tumor suppressor and deubiquitinase BAP1 promotes DNA double-strand break repair. Proc Natl Acad Sci U S A. 2014;111(1):285–90.

6. Zhao W, Steinfeld JB, Liang F, Chen X, Maranon DG, Jian Ma C, et al. BRCA1-BARD1 promotes RAD51-mediated homologous DNA pairing. Nature [Internet]. 2017;550(7676):360–5. Available from: http://dx.doi.org/10.1038/nature24060

7. Patel DS, Misenko SM, Her J, Bunting SF. BLM helicase regulates DNA repair by counteracting RAD51 loading at DNA double-strand break sites. J Cell Biol. 2017;216(11):3521–34.

8. Michl J, Zimmer J, Tarsounas M. Interplay between Fanconi anemia and homologous recombination pathways in genome integrity. EMBO J. 2016;35(9):909–23.

9. Dubbury SJ, Boutz PL, Sharp PA. CDK12 regulates DNA repair genes by suppressing intronic polyadenylation. Nature [Internet]. 2018;564(7734):141–5. Available from: http://dx.doi.org/10.1038/s41586-018-0758-y

10. Zannini L, Delia D, Buscemi G. CHK2 kinase in the DNA damage response and beyond. J Mol Cell Biol. 2014;6(6):442–57.

11. Ma L, Westbroek A, Jochemsen AG, Weeda G, Bosch A, Bootsma D, et al. Mutational analysis of ERCC3, which is involved in DNA repair and transcription initiation: identification of domains essential for the DNA repair function. Mol Cell Biol. 1994;14(6):4126–34.

12. Castillo A, Paul A, Sun B, Huang TH, Wang Y, Yazinski SA, et al. The BRCA1-interacting protein Abraxas is required for genomic stability and tumor suppression. Cell Rep [Internet]. 2014;8(3):807–17. Available from: http://dx.doi.org/10.1016/j.celrep.2014.06.050

13. Swuec P, Renault L, Borg A, Shah F, Murphy VJ, van Twest S, et al. The FA Core Complex Contains a Homo-dimeric Catalytic Module for the Symmetric Mono-ubiquitination of FANCI-FANCD2. Cell Rep [Internet]. 2017;18(3):611–23. Available from: http://dx.doi.org/10.1016/j.celrep.2016.11.013

14. Schmidt DR, Schreiber SL. Molecular association between ATR and two components of the nucleosome remodeling and deacetylating complex, HDAC2 and CHD4. Biochemistry. 1999;38(44):14711–7.

15. Korhonen Mari K., Vuorenmaa E, Nyström M. The First Functional Study of MLH3 Mutations Found in Cancer Patients. Genes Chromosomes Cancer. 2008;47(Septemer):804–9.

16. Kalev P, Simicek M, Vazquez I, Munck S, Chen L, Soin T, et al. Loss of PPP2R2A inhibits homologous recombination DNA repair and predicts tumor sensitivity to PARP inhibition. Cancer Res. 2012;72(24):6414–24.

17. Nowacka-Zawisza M, Wis̈nik E, Wasilewski A, Skowrońska M, Forma E, Brys̈ M, et al. Polymorphisms of Homologous Recombination RAD51, RAD51B, XRCC2, and XRCC3 Genes and the Risk of Prostate Cancer. Anal Cell Pathol. 2015;2015.

18. Menon V, Povirk L. Involvement of p53 in the repair of DNA double strand breaks: Multifaceted roles of p53 in homologous recombination repair (HRR) and non-homologous end joining (NHEJ). Subcell Biochem. 2014;85(380):321–36.

19. Polaczek R, Schürmann P, Speith LM, Geffers R, Dürst M, Hillemanns P, et al. Germline variation of Ribonuclease H2 genes in ovarian cancer patients. J Ovarian Res. 2020;13(1):4–11.

20. Zimmer K, Puccini A, Xiu J, Baca Y, Spizzo G, Lenz HJ, et al. WRN-mutated colorectal cancer is characterized by a distinct genetic phenotype. Cancers (Basel). 2020;12(5):1–10.

**Supplementary Tab. S2**: Summary of all mutations detected by the HRD panel

| **Probe** | **Gen** | **HGVS c.** | **HGVS p.** | **allele frequency** |
| --- | --- | --- | --- | --- |
| PC_3 | CDK12 | NM_016507.3:c.1056del | NP_057591.2:p.Met353fs | 68,07 |
| PC_4 | RAD54L | NM_003579.3:c.2027_2028del | NP_003570.2:p.His676fs | 43,30 |
|  | CDK12 | NM_016507.3:c.1284_1288del | NP_057591.2:p.Lys430fs | 19,27 |
|  | CDK12 | NM_016507.3:c.2846+1G>T |  | 57,73 |
| PC_5 | TP53 | NM_000546.5:c.254del | NP_000537.3:p.Pro85fs | 74,36 |
| PaC_1 | ARID1A | NM_006015.4:c.1015del | NP_006006.3:p.Ala339fs | 16,04 |
|  | ERCC3 | NM_000122.1:c.1933C>T | NP_000113.1:p.Arg645* | 27,24 |
|  | ATR | NM_001184.3:c.2028dup | NP_001175.2:p.Ile677fs | 21,84 |
|  | FANCF | NM_022725.3:c.891_892del | NP_073562.1:p.Glu298fs | 24,62 |
|  | ATM | NM_000051.3:c.5071A>C | NP_000042.3:p.Ser1691Arg | 35,85 |
|  | TP53 | NM_000546.5:c.524G>A | NP_000537.3:p.Arg175His | 22,33 |
|  | TP53 | NM_000546.5:c.267del | NP_000537.3:p.Ser90fs | 20,71 |
| PaC_2 | TP53 | NM_000546.5:c.824G>A | NP_000537.3:p.Cys275Tyr | 14,84 |
|  | TP53 | NM_000546.5:c.783-1G>T |  | 12,07 |
| PaC_3 | TP53 | NM_000546.5:c.524G>A | NP_000537.3:p.Arg175His | 36,16 |
| PaC_5 | TP53 | NM_000546.5:c.637C>T | NP_000537.3:p.Arg213* | 32,42 |
| PaC_6 | TP53 | NM_000546.5:c.406C>T | NP_000537.3:p.Gln136* | 26,35 |
| PaC_7 | ARID1A | NM_006015.4:c.252_260dup | NP_006006.3:p.Gly87_Ala88insGlyGlyGly | 23,51 |
|  | TP53 | NM_000546.5:c.712T>A | NP_000537.3:p.Cys238Ser | 22,30 |
| PaC_8 | TP53 | NM_000546.5:c.844C>T | NP_000537.3:p.Arg282Trp | 11,04 |
| PaC_9 | BAP1 | NM_004656.3:c.592dup | NP_004647.1:p.Glu198fs | 28,11 |
| BC_1 | TP53 | NM_000546.5:c.581T>C | NP_000537.3:p.Leu194Pro | 38,10 |
| BC_2 | BRCA1 | NM_007294.3:c.5266dup | NP_009225.1:p.Gln1756fs | 71,31 |
| BC_3 | BRCA2 | NM_000059.3:c.3975_3978dup | NP_000050.2:p.Ala1327fs | 65,00 |
|  | ARID1A | NM_006015.4:c.311A>C | NP_006006.3:p.Asn104Thr | 21,91 |
|  | RAD50 | NM_005732.3:c.3439A>T | NP_005723.2:p.Ile1147Leu | 50,36 |
|  | TP53 | NM_000546.5:c.376-2A>G | Splice | 42,64 |
| BC_4 | TP53 | NM_000546.5:c.637C>T | NP_000537.3:p.Arg213* | 14,29 |
|  | BRCA2 | NM_000059.3:c.5073dup | NP_000050.2:p.Trp1692fs | 18,70 |
|  | WRN | NM_000553.4:c.1489_1524del | NP_000544.2:p.Met497_Glu508del | 24,41 |
| BC_5 | TP53 | NM_000546.5:c.713G>T | NP_000537.3:p.Cys238Phe | 22,12 |
|  | BRCA1 | NM_007294.3:c.1171dup | NP_009225.1:p.Glu391fs | 56,82 |
| BC_8 | TP53 | NM_000546.5:c.637C>T | NP_000537.3:p.Arg213* | 40,78 |
| BC_10 | TP53 | NM_000546.5:c.1027G>T | NP_000537.3:p.Glu343* | 60,53 |
| BC_12 | TP53 | NM_000546.5:c.818G>A | NP_000537.3:p.Arg273His | 23,49 |
|  | ERCC3 | NM_000122.1:c.2294G>A | NP_000113.1:p.Arg765Gln | 52,61 |
| BC_14 | TP53 | NM_000546.5:c.517G>T | NP_000537.3:p.Val173Leu | 49,83 |
| BC_16 | WRN | NM_000553.4:c.3785C>G | NP_000544.2:p.Thr1262Arg | 45,72 |
|  | TP53 | NM_000546.5:c.487T>C | NP_000537.3:p.Tyr163His | 53,14 |
| BC_18 | ATR | NM_001184.3:c.2840C>T | NP_001175.2:p.Thr947Ile | 37,82 |
|  | PALB2 | NM_024675.3:c.2908C>T | NP_078951.2:p.Leu970Phe | 47,84 |
| OvCa_1 | RAD51D | NM_002878.3:c.763_764del | NP_002869.3:p.Arg255fs | 56,91 |
|  | TP53 | NM_000546.5:c.742C>T | NP_000537.3:p.Arg248Trp | 55,04 |
| OvCa_2 | RNASEH2B | NM_024570.4:c.529G>A | NP_078846.2:p.Ala177Thr | 89,61 |
|  | BLM | NM_000057.4:c.11T>C | NP_000048.1:p.Val4Ala | 30,93 |
|  | TP53 | NM_000546.5:c.814G>A | NP_000537.3:p.Val272Met | 80,49 |
|  | BRCA1 | NM_007294.3:c.160C>T | NP_009225.1:p.Gln54* | 91,08 |
| OvCa_3 | TP53 | NM_000546.5:c.978del | NP_000537.3:p.Glu326fs | 76,67 |
|  | BRCA1 | NM_007294.3:c.4485-2A>G | Splice | 52,44 |
| OvCa_4 | BRCA1 | NM_007294.3:c.3481_3491del | NP_009225.1:p.Glu1161fs | 76,66 |
|  | FANCD2 | NM_033084.6:c.2801C>G | NP_149075.2:p.Ser934Cys | 73,66 |
| OvCa_5 | BRCA2 | NM_000059.3:c.7617+2T>G | Splice | 80,85 |
|  | TP53 | NM_000546.5:c.659A>G | NP_000537.3:p.Tyr220Cys | 60,48 |
| OvCa_6 | BRCA1 | c.3700_3704del | NP_009225.1:p.Val1234fs | 92,76 |
|  | TP53 | NM_000546.5:c.823T>C | NP_000537.3:p.Cys275Arg | 92,63 |
|  | ATRX | NM_000489.6:c.6391C>T | NP_000480.3:p.Arg2131* | 43,92 |
| OvCa_7 | BRCA1 | NM_007294.3:c.5017_5019del | NP_009225.1:p.His1673del | 81,95 |
|  | TP53 | NM_000546.5:c.817C>T | NP_000537.3:p.Arg273Cys | 73,47 |
| OvCa_8 | BRCA1 | NM_007294.3:c.3354_3355del | NP_009225.1:p.Gln1118fs | 87,27 |
|  | TP53 | NM_000546.5:c.584T>A | NP_000537.3:p.Ile195Asn | 70,79 |
| OvCa_9 | MRE11 | NM_005591.3:c.530C>T | NP_005582.1:p.Ala177Val | 36,95 |
|  | TP53 | NM_000546.5:c.772G>T | NP_000537.3:p.Glu258* | 66,92 |
|  | BRCA1 | NM_007294.3:c.2222C>G | NP_009225.1:p.Ser741Cys | 18,65 |
| OvCa_10 | BLM | NM_000057.3:c.191A>T | NP_000048.1:p.Asp64Val | 30,92 |
|  | TP53 | NM_000546.5:c.614A>G | NP_000537.3:p.Tyr205Cys | 57,61 |
| OvCa_11 | ATM | NM_000051.3:c.5558A>T | NP_000042.3:p.Asp1853Val | 49,29 |
|  | BRCA1 | NM_007294.3:c.5266dup | NP_009225.1:p.Gln1756fs | 84,21 |
|  | TP53 | NM_000546.5:c.376-1G>A |  | 46,61 |
| OvCa_13 | TP53 | NM_000546.5:c.743G>A | NP_000537.3:p.Arg248Gln | 69,96 |
| OvCa_14 | BRCA2 | NM_000059.3:c.5782G>T | NP_000050.2:p.Glu1928* | 58,01 |
|  | TP53 | NM_000546.5:c.1024C>T | NP_000537.3:p.Arg342* | 69,50 |
| OvCa_15 | ATM | NM_000051.3:c.5558A>T | NP_000042.3:p.Asp1853Val | 63,47 |
|  | TP53 | NM_000546.5:c.673-2A>G |  | 43,86 |
| OvCa_16 | TP53 | NM_000546.5:c.559+1G>A |  | 57,58 |
|  | BRCA1 | NM_007294.3:c.68_69del | NP_009225.1:p.Glu23fs | 74,33 |
| OvCa_17 | TP53 | NM_000546.5:c.809T>G | NP_000537.3:p.Phe270Cys | 40,03 |
|  | BRCA1 | NM_007294.3:c.3556_3566del | NP_009225.1:p.Leu1186fs | 38,99 |
| OvCa_18 | BRCA2 | NM_000059.3:c.6306_6452del | NP_000050.2:p.Lys2104_Ser2152del | 46,47 |
|  | TP53 | NM_000546.5:c.743G>A | NP_000537.3:p.Arg248Gln | 77,36 |
| OvCa_21 | TP53 | NM_000546.5:c.515_524del | NP_000537.3:p.Val172fs | 24,82 |
| OvCa_22 | ARID1A | NM_006015.4:c.6491G>A | NP_006006.3:p.Arg2164Gln | 23,34 |
|  | WRN | NM_000553.4:c.673C>T | NP_000544.2:p.Arg225* | 65,24 |
|  | TP53 | NM_000546.5:c.742C>T | NP_000537.3:p.Arg248Trp | 58,63 |
| OvCa_23 | FANCA | NM_000135.2:c.2107C>T | NP_000126.2:p.Gln703* | 21,26 |
|  | TP53 | NM_000546.5:c.742C>T | NP_000537.3:p.Arg248Trp | 63,37 |
| OvCa_24 | NBN | NM_002485.4:c.970_971del | NP_002476.2:p.Asp324fs | 54,23 |
| OvCa_25 | ERCC3 | NM_000122.1:c.847C>T | NP_000113.1:p.Arg283Cys | 31,39 |
| OvCa_26 | ATM | NM_000051.3:c.5071A>C | NP_000042.3:p.Ser1691Arg | 87,08 |
|  | TP53 | NM_000546.5:c.637C>T | NP_000537.3:p.Arg213* | 80,25 |
| OvCa_27 | TP53 | NM_000546.5:c.824G>T | NP_000537.3:p.Cys275Phe | 92,59 |
| OvCa_28 | ATM, C11orf65 | NM_000051.3:c.7327C>T | NP_000042.3:p.Arg2443* | 57,84 |
|  | TP53 | NM_000546.5:c.594del | NP_000537.3:p.Gly199fs | 33,26 |
| OvCa_30 | WRN | NM_000553.4:c.1901G>A | NP_000544.2:p.Gly634Asp | 51,60 |
|  | TP53 | NM_000546.5:c.734G>T | NP_000537.3:p.Gly245Val | 24,17 |
| OvCa_31 | WRN | NM_000553.4:c.1717A>G | NP_000544.2:p.Thr573Ala | 63,39 |
|  | TP53 | NM_000546.5:c.394A>G | NP_000537.3:p.Lys132Glu | 74,37 |
| OvCa_32 | TP53 | NM_000546.5:c.843C>G | NP_000537.3:p.Asp281Glu | 79,20 |
|  | BRIP1 | NM_032043.2:c.1224_1225del | NP_114432.2:p.Tyr408fs | 92,06 |
| OvCa_33 | TP53 | NM_000546.5:c.742C>T | NP_000537.3:p.Arg248Trp | 64,06 |
| OvCa_34 | ATR | NM_001184.3:c.6892G>A | NP_001175.2:p.Asp2298Asn | 53,81 |
|  | FANCI | NM_018193.2:c.1810A>G | NP_060663.2:p.Met604Val | 52,65 |
| OvCa_36 | ARID1A | NM_006015.4:c.1210C>T | NP_006006.3:p.Gln404* | 64,86 |
|  | ATM | NM_000051.3:c.3275C>G | NP_000042.3:p.Ser1092* | 65,90 |
| OvCa_37 | RAD50 | NM_005732.3:c.463A>G | NP_005723.2:p.Ile155Val | 42,51 |
|  | TP53 | NM_000546.5:c.742C>T | NP_000537.3:p.Arg248Trp | 22,53 |
| OvCa_38 | TP53 | NM_000546.5:c.742C>T | NP_000537.3:p.Arg248Trp | 30,12 |
| OvCa_39 | TP53 | NM_000546.5:c.692_693insGG | NP_000537.3:p.Ile232fs | 38,97 |
| OvCa_40 | TP53 | NM_000546.5:c.380C>T | NP_000537.3:p.Ser127Phe | 70,74 |
| OvCa_41 | TP53 | NM_000546.5:c.581T>G | NP_000537.3:p.Leu194Arg | 58,41 |
| OvCa_42 | TP53 | NM_000546.5:c.469G>T | NP_000537.3:p.Val157Phe | 73,88 |
| OvCa_43 | TP53 | NM_000546.5:c.880del | NP_000537.3:p.Glu294fs | 48,85 |
| OvCa_44 | TP53 | NM_000546.5:c.536A>G | NP_000537.3:p.His179Arg | 70,62 |
| OvCa_45 | TP53 | NM_000546.5:c.916C>T | NP_000537.3:p.Arg306* | 33,40 |
| OvCa_47 | TP53 | NM_000546.5:c.833C>G | NP_000537.3:p.Pro278Arg | 30,00 |
| OvCa_48 | TP53 | NM_000546.5:c.841G>A | NP_000537.3:p.Asp281Asn | 43,54 |
| OvCa_49 | TP53 | NM_000546.5:c.844C>T | NP_000537.3:p.Arg282Trp | 53,69 |
| OvCa_50 | TP53 | NM_000546.5:c.405C>G | NP_000537.3:p.Cys135Trp | 79,70 |
| OvCa_51 | TP53 | NM_000546.5:c.523C>G | NP_000537.3:p.Arg175Gly | 76,56 |
| OvCa_52 | TP53 | NM_000546.5:c.818G>A | NP_000537.3:p.Arg273His | 42,29 |
| OvCa_54 | TP53 | NM_000546.5:c.584T>C | NP_000537.3:p.Ile195Thr | 53,87 |
| OvCa_56 | TP53 | NM_000546.5:c.743G>A | NP_000537.3:p.Arg248Gln | 33,75 |
|  | FANCA | NM_000135.2:c.1340C>T | NP_000126.2:p.Ser447Leu | 62,36 |
| OvCa_58 | ATM | NM_000051.3:c.7814T>G | NP_000042.3:p.Ile2605Arg | 20,86 |
|  | TP53 | NM_000546.5:c.820G>T | NP_000537.3:p.Val274Phe | 56,37 |
| OvCa_59 | TP53 | NM_000546.5:c.993+1G>A |  | 78,71 |
| OvCa_60 | MRE11 | NM_005591.3:c.1462C>T | NP_005582.1:p.Arg488Cys | 48,04 |
|  | FANCG | NM_004629.1:c.1382A>C | NP_004620.1:p.Gln461Pro | 50,98 |
| OvCa_61 | TP53 | NM_000546.5:c.434T>A | NP_000537.3:p.Leu145Gln | 70,55 |
| OvCa_62 | WRN | NM_000553.4:c.3495G>T | NP_000544.2:p.Gln1165His | 43,73 |
| OvCa_63 | BRCA2 | NM_000059.3:c.755_758del | NP_000050.2:p.Asp252fs | 91,87 |
|  | TP53 | NM_000546.5:c.841G>A | NP_000537.3:p.Asp281Asn | 90,78 |
| OvCa_64 | BRCA2 | NM_000059.3:c.7612A>T | NP_000050.2:p.Lys2538* | 62,00 |
|  | TP53 | NM_000546.5:c.731G>A | NP_000537.3:p.Gly244Asp | 58,56 |
| OvCa_65 | ATM | NM_000051.3:c.7390T>C | NP_000042.3:p.Cys2464Arg | 87,59 |
|  | BRCA1 | NM_007294.3:c.3006delC | NP_009225.1:p.Phe1003fs | 77,53 |
|  | TP53 | NM_000546.5:c.422G>A | NP_000537.3:p.Cys141Tyr | 72,95 |
|  | FANCL | c.427_450delinsTT | NP_060532.2:p.Ala143fs | 28,29 |

**Supplementary Fig. S1**


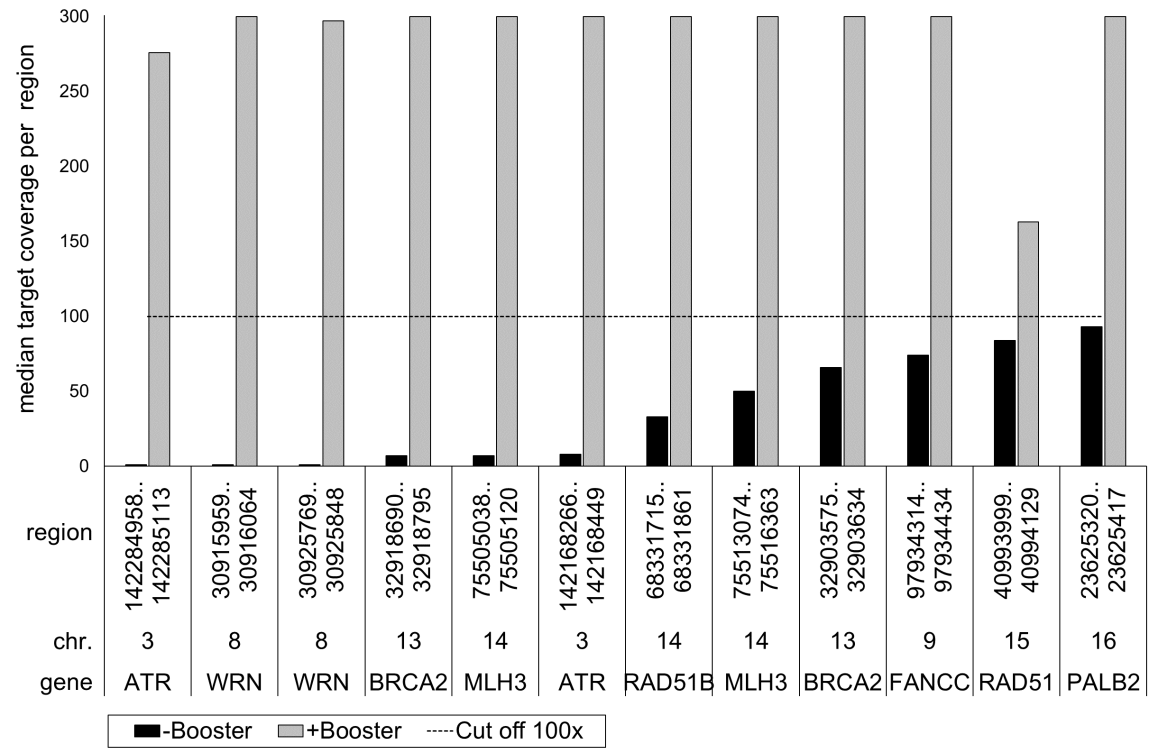


**Supplementary Fig. 1**: Median coverage of specific regions with and without booster panel.

Sequencing with the original HRD panel (-Booster) manifested in an insufficient coverage below the cut off at 100 reads per target in 12 areas (black bars). With the additional use of the booster panel (+Booster), the coverage improved for those areas (grey bars). For coverage analysis, 15 training cohort samples were used. Values greater than 300x is set to 300 by default and therefore not shown.

**Supplementary Fig. S2**


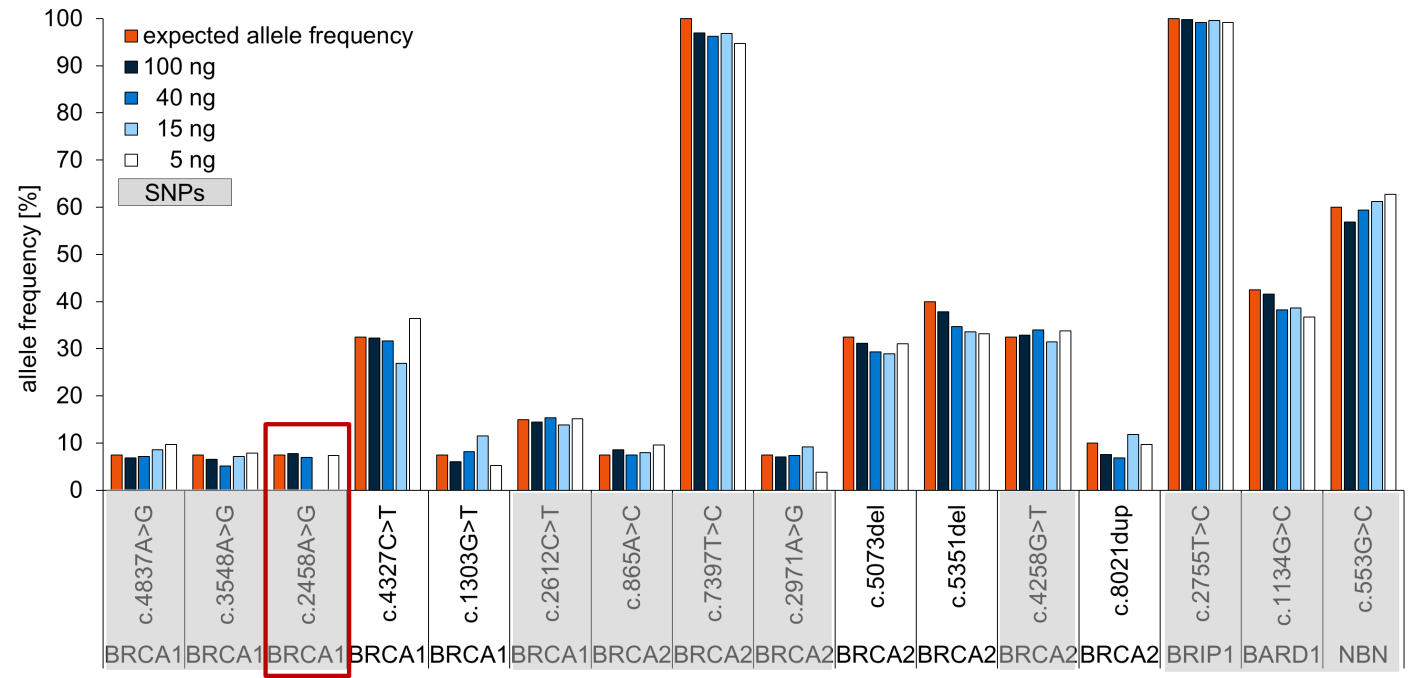


**Supplementary Fig.2**: Variant detection with decreasing amounts of DNA input

Different amounts of gDNA (100, 40, 15 and 5 ng) of the *BRCA* Somatic Multiplex FFPE standard were used for library preparation. All given variants were detectable by means of the HRD panel in similar allele frequencies (one exception with DNA input of 15 ng, marked in red) compared to manufactures information. All grey columns represent single nucleotide polymorphisms (SNPs), which were automatically dismissed by filtering processes.

**Supplementary Fig. S3**

**
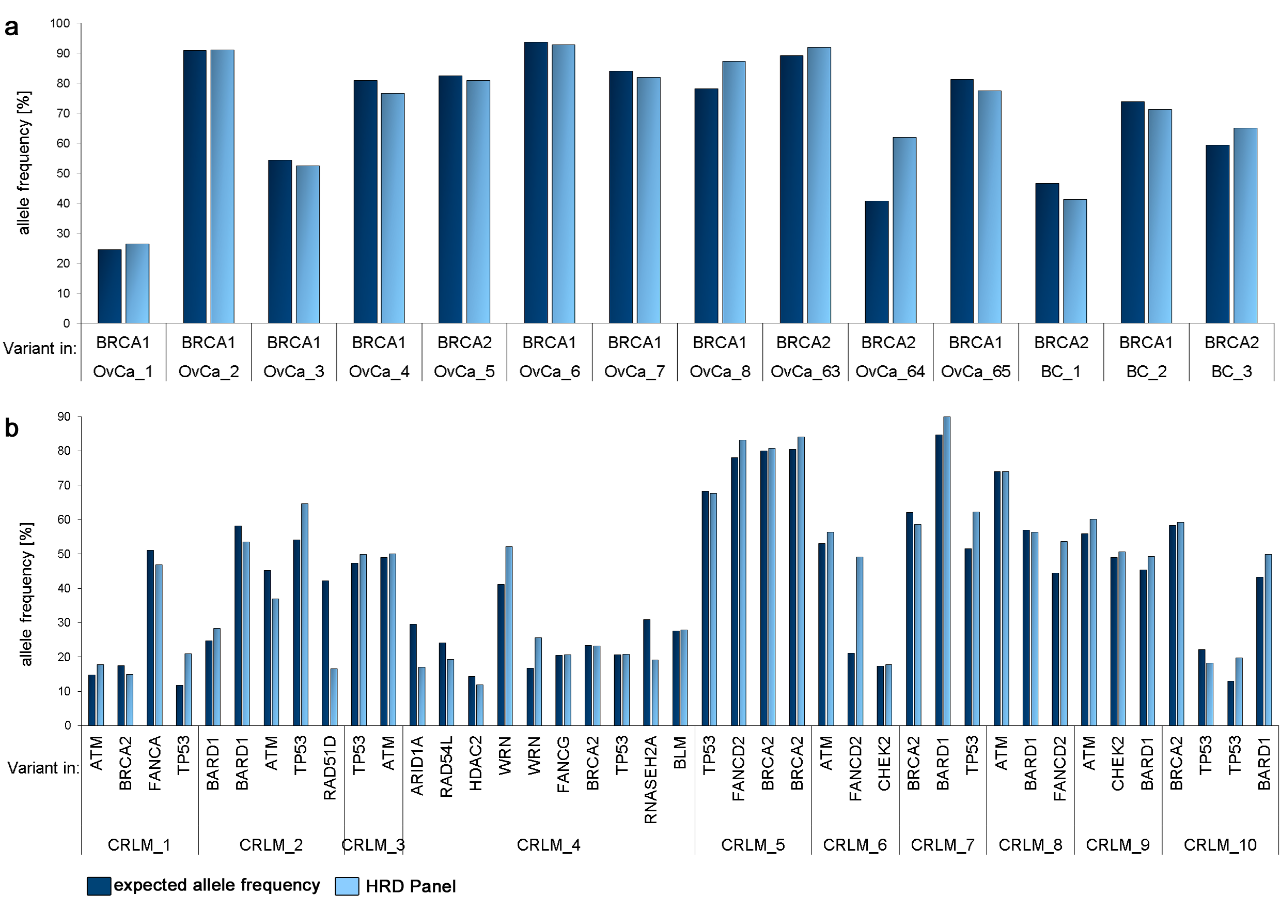
**

**Supplementary Fig.3**: Validation of HRD panel performance using samples with known mutations.

*BRCA1/2* mutations of ovarian (OvCa) and breast cancer (BC) samples were initially sequenced by a *BRCA*-specific NGS panel. The same samples were now sequenced with the new HRD panel (a). Samples of colorectal liver metastases (CRLM) were previously processed by whole exome sequencing (WES) (b). In both cases, the new HRD Panel showed complete concordance throughout all samples.
